# Statistical analysis for masked hybrid system lifetime data in step-stress partially accelerated life test with progressive hybrid censoring

**DOI:** 10.1371/journal.pone.0186417

**Published:** 2017-10-23

**Authors:** Xiaolin Shi, Yanchao Liu, Yimin Shi

**Affiliations:** 1 School of Electronics Engineering, Xi’an University of Posts and Telecommunications, Xi’an, China; 2 Department of Applied Mathematics, Northwestern Polytechnical University, Xi’an, China; State University of New York, UNITED STATES

## Abstract

In this paper, we investigate a step-stress partially accelerated lifetime test for the four-component hybrid systems with Type-II progressive hybrid censoring scheme while the life time of system component follows exponential failure rate. In many cases, the exact component causing the system failure cannot be identified and the cause of failure is masked. Based on Type-II progressively hybrid censored and masked data, the maximum likelihood estimations for unknown parameters and acceleration factor are obtained. In addition, approximate confidence interval and bootstrap confidence interval are presented by using the asymptotic distributions of the maximum likelihood estimations for unknown parameters and bootstrap method, respectively. Finally, the proposed method is illustrated through the simulation studies.

## Introduction

The failure data from multi-component systems plays an important role in system reliability analysis. Usually, the system failure data contain the failure time and the information on the exact component causing the system failure. In some cases, however, due to lack of proper diagnostic equipment or cost and time constraints, the exact component causing the system failure is not identified, and the failure cause is isolated to a subset of the system components. Such type of data is called masked data. Recently, the statistical analysis for masked data has been studied by several authors. Usher and Hodgson [1] initially proposed the masked data, and derived the maximum likelihood estimations (MLE) for unknown parameter in a series system. Lin [2] presented the Bayes estimation for a two-component series system with exponential components. Sarhan [3] focused on two and three component series system when component life follows Weibull distribution, and obtained the MLE of unknown parameter. Bayesian analysis for the two-component series system with Pareto components were discussed in [4]. The MLE and Bayesian estimation (BE) were presented for parallel system in [5–6]. When system component lifetime follows constant and linear failure rate, the MLE and other estimation methods were studied in [7–9]. Jiang [10] presented the MLE and BE for a series system under exponential failure rate. The MLE and approximate confidence interval were derived for a three-component hybrid systems in [11].

For some products with high reliability and long lifetime, it is difficult to get the failure information under use stress level. Accelerated life test (ALT) is a strategy to overcome the problem. Based on the failure data of accelerated life test to analyze the life characteristics of product, we need to use the relationship between the reliability index and stress levels, that acceleration model. In many cases, however, the accelerated model does not exist or is difficult to find so that ALT is not available. Partially accelerated life test (PALT) is a better way to use in practical application. A class of PALT is step-stress partially accelerated life test (SSPALT). In SSPALT, a test product runs at use stress level first and if it does not fail in a prefixed time point, and then it run at accelerated stress level until failures or the observations are censored. Based on Type-I censoring scheme, reliability analysis for a series system in SSPALT was studied in [12], where the components lifetime follows Weibull distributions. Ismail [13] presented the MLE and interval estimation of the generalized exponential distribution parameters under SSPALT with Type-II censoring. Sun [14] made inference for Burr-XII distribution parameters in SSPALT, and a hybrid algorithm combined Gibbs sampling and Metropolis-Hastings sampling was used. The MLE, approximate confidence interval (ACI) and Bootstrap confidence intervals **(**BCI) were given under SSPALT with Type-I censoring in [15]. Ref. [16] considered the MLE and ACI in SSPALT for the Burr-XII distribution with Type-I censoring. Ref. [17] proposed Bayesian analysis for the series system under SSPALT.

In SSPALT, it is necessary to take progressively hybrid censoring schemes into account. Type-II progressively hybrid censoring (PHC), initially proposed by Kundu D [18], is a new censoring scheme. It can not only remove the tested product to study properties during the test, but also effectively control the test of time and reduce test costs. Therefore, the censoring scheme has attracted wide attention. SSPALT was studied under Type-I PHC in [19–20]. Ismail [21] made inference for a Weibull distribution in SSPALT with an adaptive Type-I progressively hybrid censored data. Some related literatures are [22–25]. So far, most researches for masked data focused on a system that is either series or parallel only. In many real situations, however, it is often seen that a system functions in a way better described by a combination of series and parallel constructions. For example, computer network transmission systems, power transmission systems in power stations, and complex electric burst networks [26]. Statistical analysis on hybrid system with masked data was very rare. Wang *et al*. [11] studied parameter inference on the independent component hybrid system with masked data, where the component's failure rate was considered as a constant or a linear function. Assuming dependent lifetimes of components modelled by Marshall and Olkin’s bivariate exponential distribution in the system, Sha *et al*. [27] investigated statistical inference on dependent component hybrid system with masked data. In addition to constant and the linear failure rates, the exponential failure rate is also worthy of attention and are studied [28]. Statistical inference for a series system was discussed based on complete samples in [28], where the component's failure rate was considered as an exponential function. However, they did not involve accelerated life testing and PHC schemes. Different from previous research, this paper mainly discussed SSPALT for four-component hybrid system with masked data under PHC when the component's failure rate was exponential function.

The rest of this paper is organized as follows. A brief description of the SSPALT model and the assumptions are elaborated in Section 2. MLEs for unknown parameters and acceleration factor are presented in Section 3. Approximate confidence interval and Bootstrap confidence interval for unknown parameters and acceleration factor are obtained in section 4. In Section 5, the Monte-Carlo simulation studies are carried out for different sample sizes and for different progressive censoring schemes. Some conclusions are presented in Section 6.

## Model description and basic assumptions

**Fig 1:**
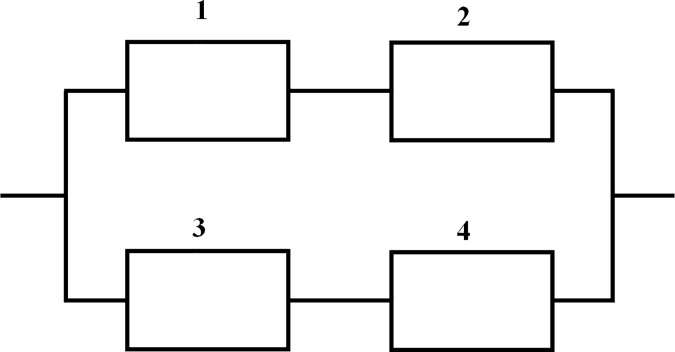
Hybrid system (a).

**Fig 2:**
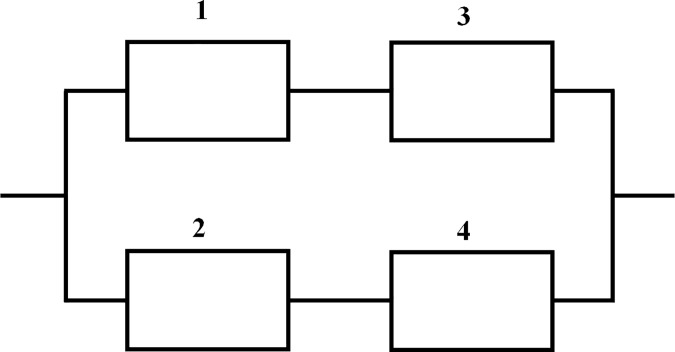
Hybrid system (b).

Two fundamental types of hybrid systems with four components is discussed in this paper, which is shown in Fig 1 and Fig 2.

For above two fundamental types of hybrid systems, the SSPALT model under Type-II PHC is described as follows.

### Model description

At the start, *n* identical systems are placed on test at use stress level *s*^0^. Each of these systems has four components. At the first failure time *t*_1_, *R*_1_ systems are randomly removed from the remaining *n*−1 systems. At the second failure time *t*_2_, *R*_2_ systems are randomly removed from the remaining *n*−*R*_1_−2 systems, and so on. At time *τ*, all of the surviving systems are moved to accelerated stress level *s*^1^ (*s*^1^ > *s*^0^). If the *m*th failure time *t*_*m*_ occurs before the preset time point *η* (*η* > *τ*), then the test terminates at the time point *t*_*m*_ and all of the remaining *R*_*m*_ = *n*−*m*−(*R*_1_ + ⋯ + *R*_*m*−1_) are removed, where *τ*, *R*_1_,*R*_2_⋯,*R*_*m*_ are prefixed and *m* < *n*. Otherwise, If the failure time *t*_*m*_ does not occur before the time point *η*, and only *d* failure occurs before the time point *η*, where *d* < *m*, then all the remaining $R_{d}^{*}$ systems are removed at the time point *η* and test terminates, where $R_{d}^{*}=n-d-(R_{1}+\cdots +R_{d})$. Let *n*_*u*_ denote the number of systems failure at use stress level, *n*_*v*_ be the number of systems failure at accelerated stress level, and *R* be the whole number of systems failure at the life test, where *R* = *n*_*u*_ + *n*_*v*_. Assume the observed system failure times can be obtained, and the cause of system failure is only known to belong to the minimum random subset of the set {1,2,3,4}. Let *S*_*i*_ be the set of components causing the system *i* failure, *s*_*i*_ represent the observations of *S*_*i*_, Then *s*_*i*_ ⊆ {{1},{2},{3},{4},{1,2},{1,3},{1,4},{2,3}{2,4},{3,4},{1,2,3},{1,3,4}{2,3,4}{1,3,4},{1,2,3,4}} Thus, the available lifetime data of the hybrid system can be obtained as follows:

Case Ⅰ: $\left\{(t_{1},s_{1}),(t_{2},s_{2}),\cdots ,(t_{n_{u}},s_{n_{u}}),\cdots ,(t_{m},s_{m})\right\}$, if *τ* < *t*_*m*_ ≤ *η*,
Case Ⅱ: $\left\{(t_{1},s_{1}),(t_{2},s_{2}),\cdots ,(t_{n_{u}},s_{n_{u}}),\cdots ,(t_{n_{u}+n_{v}},s_{n_{u}+n_{v}})\right\}$, if *t*_*m*_ > *η*.

### Basic assumptions

In order to analyze the lifetime data of the hybrid system from SSPALT, we make the following assumptions.

A1. At use stress level *s*^0^, the lifetime of the *j*-th component in the hybrid system follows exponential failure rate:

$$h_{1j}(t)=\lambda_{j}e^{t},t>0,\lambda_{j}>0,j=1,2,3,4.$$ (1)

Then the cumulative distribution function (CDF), probability density function (PDF) and reliability function (RF) for the *j*-th component of the system are given (*j* = 1,2,3,4), respectively.

$$F_{1j}\left(t\right)=1-\exp\left[-\lambda_{j}\left(e^{t}-1\right)\right],\;t>0,\lambda_{j}>0,$$ (2)

$$f_{1j}\left(t\right)=\lambda_{j}e^{t}\cdot \exp\left[-\lambda_{j}\left(e^{t}-1\right)\right],\;t>0,\lambda_{j}>0,$$ (3)

$${\bar{F}}_{1j}\left(t\right)=\exp\left[-\lambda_{j}\left(e^{t}-1\right)\right],\;t>0,\lambda_{j}>0.$$ (4)

A2. The tampered random variable (TRV) model holds:

$$T=X,\;\mathrm{if}\;X\leq \tau ;\;T=\tau +\alpha^{-1}(X-\tau ),\;\mathrm{if}\;X>\tau ,$$ (5)

where *T* denotes the total lifetime of a component and *X* is the lifetime of the system at use stress level, *τ* is the stress change time and *α* is the acceleration factor, *α* > 1, see [29]. From Eqs (1–5), at accelerated stress level *s*^1^, the CDF, FRF, RF and PDF for the *j*-th (*j* = 1,2,3,4) component of the system are given by

$$\begin{array}{l} F_{2j}\left(t\right)=1-\exp\left[-\lambda_{j}\left(e^{\left(\tau +\alpha (t-\tau )\right)}-1\right)\right],\;h_{2j}(t)=a\lambda_{j}e^{\left(\tau +\alpha (t-\tau )\right)},\;{\bar{F}}_{2j}\left(t\right)=\exp\left[-\lambda_{j}\left(e^{\left(\tau +\alpha (t-\tau )\right)}-1\right)\right],\;t>0,\lambda_{j}>0,\\ \;f_{2j}\left(t\right)=a\lambda_{j}e^{\left(\tau +\alpha (t-\tau )\right)}\cdot \exp\left[-\lambda_{j}\left(e^{\left(\tau +\alpha (t-\tau )\right)}-1\right)\right],\;t>0,\lambda_{j}>0. \end{array}$$ (6)

A3. The lifetime of tested systems is independent and identically distributed at both use stress level and accelerated stress level.

A4. Masking is independent of the cause of system failure, the failure time and stress level.

## Maximum likelihood estimations

Assume *T*_*i*_ is the lifetime of the system *i*, *t*_*i*_ denote observations of *T*_*i*_. The lifetime of the *j*-th component of the system *i* are denoted by *T*_*ij*_, the observations of *T*_*ij*_ is expressed as *t*_*ij*_, *i* = 1,2,⋯,*n*, *j* = 1,2,3,4. Note that *S*_*i*_ be the set of components causing the system *i* failure, *s*_*i*_ represent the observations of *S*_*i*_. If *s*_*i*_ include more than one element, then the cause of the system failure is not exact and it is masked. Let *K*_*i*_ denote the specific component inducing the system *i* failure. Unless otherwise noted, *k* = 1,2 respectively denote the use stress level and accelerated stress level in this paper. So, when *k* = 1, *i* = 1,⋯,*n*_*u*_ and when *k* = 2, *i* = *n*_*u*_+1,⋯,*R*.

Now, let us consider the system *i*, where *i* = 1,⋯,*n*_*u*_; *k* = 1 or *i* = *n*_*u*_+1,⋯,*R*; *k* = 2.

$$\begin{array}{l} P\left(t_{i}<T_{i}<t_{i}+dt_{i},S_{i}=s_{i}\right)=\sum_{j=1}^{4}P\left(t_{i}<T_{i}<t_{i}+dt_{i},S_{i}=s_{i},K_{i}=j\right)\\ \;=\sum_{j=1}^{4}P\left(t_{i}<T_{i}<t_{i}+dt_{i},K_{i}=j\right)P\left(S_{i}=s_{i}|t_{i}<T_{i}<t_{i}+dt_{i},K_{i}=j\right)\\ \;=\sum_{j\in s_{i}}P\left(t_{i}<T_{i}<t_{i}+dt_{i},K_{i}=j\right)P\left(S_{i}=s_{i}|t_{i}<T_{i}<t_{i}+dt_{i},K_{i}=j\right), \end{array}$$ (7)

where *P*(*S*_*i*_ = *s*_*i*_|*t*_*i*_ < *T*_*i*_ < *t*_*i*_ + *dt*_*i*_, *K*_*i*_ = *j*) is called masked probability, and from basic assumptions A4, we derived *P*(*S*_*i*_ = *s*_*i*_|*t*_*i*_ < *T*_*i*_ < *t*_*i*_ + *dt*_*i*_, *K*_*i*_ = *j*) = *P*(*S*_*i*_ = *s*_*i*_) = *θ*_*i*_, where *P*(*t*_*i*_ < *T*_*i*_ < *t*_*i*_ + *dt*_*i*_, *K*_*i*_ = *j*) is the probability of system failure caused by component *j* at the time *t*_*i*_.

For the hybrid systems (a), the lifetime of the system *i* is

$$T_{i}=\max\left[\min\left(T_{i1},T_{i2}\right),\min\left(T_{i3},T_{i4}\right)\right].$$

The probability of the system *i* failure at the time *t*_*i*_ due to each component is presented as follows:

$$\begin{array}{l} P_{i1}=P\left(t_{i}<T_{i}<t_{i}+dt_{i},K_{i}=1\right)\\ \;=P\left(t_{i}<T_{i1}<t_{i}+dt_{i}\right)P\left(T_{i2}>t_{i}\right)\left[1-P\left(T_{i3}>t_{i}\right)P\left(T_{i4}>t_{i}\right)\right]\\ \;=f_{k1}\left(t_{i}\right){\bar{F}}_{k2}\left(t_{i}\right)\left[1-{\bar{F}}_{k3}\left(t_{i}\right){\bar{F}}_{k4}\left(t_{i}\right)\right]dt_{i}, \end{array}$$

$$\begin{array}{l} P_{i2}=P\left(t_{i}<T_{i}<t_{i}+dt_{i},K_{i}=2\right)\\ \;=P\left(T_{i1}>t_{i}\right)P\left(t_{i}<T_{i2}<t_{i}+dt_{i}\right)\left[1-P\left(T_{i3}>t_{i}\right)P\left(T_{i4}>t_{i}\right)\right]\\ \;={\bar{F}}_{k1}\left(t_{i}\right)f_{k2}\left(t_{i}\right)\left[1-{\bar{F}}_{k3}\left(t_{i}\right){\bar{F}}_{k4}\left(t_{i}\right)\right]dt_{i}, \end{array}$$

$$\begin{array}{l} P_{i3}=P\left(t_{i}<T_{i}<t_{i}+dt_{i},K_{i}=3\right)\\ \;=\left[1-P\left(T_{i1}>t_{i}\right)P\left(T_{i2}>t_{i}\right)\right]P\left(t_{i}<T_{i3}<t_{i}+dt_{i}\right)P\left(T_{i4}>t_{i}\right)\\ \;=\left[1-{\bar{F}}_{k1}\left(t_{i}\right){\bar{F}}_{k2}\left(t_{i}\right)\right]f_{k3}\left(t_{i}\right){\bar{F}}_{k4}\left(t_{i}\right)dt_{i}, \end{array}$$

$$\begin{array}{l} P_{i4}=P\left(t_{i}<T_{i}<t_{i}+dt_{i},K_{i}=4\right)\\ \;=\left[1-P\left(T_{i1}>t_{i}\right)P\left(T_{i2}>t_{i}\right)\right]P\left(T_{3}>t_{i}\right)P\left(t_{i}<T_{i4}<t_{i}+dt_{i}\right)\\ \;=\left[1-{\bar{F}}_{k1}\left(t_{i}\right){\bar{F}}_{k2}\left(t_{i}\right)\right]{\bar{F}}_{k3}\left(t_{i}\right)f_{k4}\left(t_{i}\right)dt_{i}. \end{array}$$

To ease notation, we denote

$$\begin{array}{l} f_{i1}=f_{k1}\left(t_{i}\right){\bar{F}}_{k2}\left(t_{i}\right)\left[1-{\bar{F}}_{k3}\left(t_{i}\right){\bar{F}}_{k4}\left(t_{i}\right)\right],\;f_{i2}={\bar{F}}_{k1}\left(t_{i}\right)f_{k2}\left(t_{i}\right)\left[1-{\bar{F}}_{k3}\left(t_{i}\right){\bar{F}}_{k4}\left(t_{i}\right)\right],\\ \;f_{i3}=\left[1-{\bar{F}}_{k1}\left(t_{i}\right){\bar{F}}_{k2}\left(t_{i}\right)\right]f_{k3}\left(t_{i}\right){\bar{F}}_{k4}\left(t_{i}\right),\;f_{i4}=\left[1-{\bar{F}}_{k1}\left(t_{i}\right){\bar{F}}_{k2}\left(t_{i}\right)\right]{\bar{F}}_{k3}\left(t_{i}\right)f_{k4}\left(t_{i}\right), \end{array}$$ (8)

where *i* = 1,⋯,*n*_*u*_; *k* = 1 or *i* = *n*_*u*_+1,⋯,*R*; *k* = 2. That is, if the system *i* fails at the use tress level, then *k* = 1; system *i* fails at the accelerating stress level, then *k* = 2.

Suppose the lifetime of component 1, 2, 3 and 4 in the system are *X*,*Y*,*Z*,*K*, and *X*,*Y*,*Z*,*K* are independent of each other. At use stress level, they have the same failure rate function *h*_1_(*t*) = *λe*^*t*^,*t*>0,*λ*>0. Then the system life *W* = max[min(*X*,*Y*),min(*Z*,*K*)]. According to basic assumptions A1 and A2, the reliability function of system at use stress level is obtained

$${\bar{F}}_{1}(t)=\exp\left(-2\lambda \left(e^{t}-1\right)\right)\left[2-\exp\left(-2\lambda \left(e^{t}-1\right)\right)\right],\;t>0,\lambda >0.$$

The reliability function of system at accelerated stress level is

$${\bar{F}}_{2}\left(t\right)=\exp\left(-2\lambda \left(e^{{}^{\left(\tau +\alpha (t-\tau )\right)}}-1\right)\right)\left[2-\exp\left(-2\lambda \left(e^{{}^{\left(\tau +\alpha (t-\tau )\right)}}-1\right)\right)\right],\;t>0,\lambda >0.$$

Based on Type-II PHC sample, the likelihood function for the hybrid systems (a) is given by

$$\begin{array}{l} L\left(\lambda ,a\right)\propto \prod_{i=1}^{n_{u}}\left\{\left(\sum_{j\in s_{i}}\theta_{i}f_{ij}\right)\cdot {\left[{\bar{F}}_{1}\left(t_{i}\right)\right]}^{R_{i}}\right\}\prod_{i=n_{u}+1}^{R}\left\{\left(\sum_{j\in s_{i}}\theta_{i}f_{ij}\right)\cdot {\left[{\bar{F}}_{2}\left(t_{i}\right)\right]}^{R_{i}}\right\}\cdot {\left[{\bar{F}}_{2}\left(\eta \right)\right]}^{R^{*}}\\ \;\propto \prod_{i=1}^{n_{u}}\left\{\left(\sum_{j\in s_{i}}f_{ij}\right)\cdot {\left[{\bar{F}}_{1}\left(t_{i}\right)\right]}^{R_{i}}\right\}\prod_{i=n_{u}+1}^{R}\left\{\left(\sum_{j\in s_{i}}f_{ij}\right)\cdot {\left[{\bar{F}}_{2}\left(t_{i}\right)\right]}^{R_{i}}\right\}\cdot {\left[{\bar{F}}_{2}\left(\eta \right)\right]}^{R^{*}} \end{array}$$

In the above formula, for Case Ⅰ, *R* = *m*, R^*^ = 0; For Case Ⅱ: *R* = *d*, R^*^ = *n*−*d*−(*R*_1_ +⋯+ *R*_*d*_).

The likelihood function for the hybrid systems (a) is written as:

$$\begin{array}{l} L\left(\lambda ,a\right)\propto \prod_{i=1}^{n_{u}}\left\{\left(\sum_{j\in s_{i}}f_{ij}\right)\cdot {\left[{\bar{F}}_{1}\left(t_{i}\right)\right]}^{R_{i}}\right\}\prod_{i=n_{u}+1}^{R}\left\{\left(\sum_{j\in s_{i}}f_{ij}\right)\cdot {\left[{\bar{F}}_{2}\left(t_{i}\right)\right]}^{R_{i}}\right\}\cdot {\left[{\bar{F}}_{2}\left(\eta \right)\right]}^{R^{*}}\\ \;\propto \prod_{i=1}^{R_{1}}\left\{\sum_{j\in s_{i}}f_{ij}\right\}\prod_{i=R_{1}+1}^{R_{1}+R_{2}}\left\{\sum_{j\in s_{i}}f_{ij}\right\}\prod_{i=R_{1}+R_{2}+1}^{R_{1}+R_{2}+R_{3}}\left\{\sum_{j\in s_{i}}f_{ij}\right\}\prod_{i=R_{1}+R_{2}+R_{3}+1}^{R_{1}+R_{2}+R_{3}+R_{4}}\left\{\sum_{j\in s_{i}}f_{ij}\right\}\prod_{i=R_{1}+R_{2}+R_{3}+R_{4}+1}^{R_{1}+\cdots +R_{5}}\left\{\sum_{j\in s_{i}}f_{ij}\right\}\\ \;\times \prod_{i=R_{1}+\cdots +R_{5}+1}^{R_{1}+\cdots +R_{6}}\left\{\sum_{j\in s_{i}}f_{ij}\right\}\prod_{i=R_{1}+\cdots +R_{6}+1}^{R_{1}+\cdots +R_{7}}\left\{\sum_{j\in s_{i}}f_{ij}\right\}\prod_{i=R_{1}+\cdots +R_{7}+1}^{R_{1}+\cdots +R_{8}}\left\{\sum_{j\in s_{i}}f_{ij}\right\}\prod_{i=R_{1}+\cdots +R_{8}+1}^{R_{1}+\cdots +R_{9}}\left\{\sum_{j\in s_{i}}f_{ij}\right\}\\ \;\times \prod_{i=R_{1}+\cdots +R_{9}+1}^{R_{1}+\cdots +R_{10}}\left\{\sum_{j\in s_{i}}f_{ij}\right\}\prod_{i=R_{1}+\cdots +R_{10}+1}^{R_{1}+\cdots +R_{11}}\left\{\sum_{j\in s_{i}}f_{ij}\right\}\prod_{i=R_{1}+\cdots +R_{11}+1}^{R_{1}+\cdots +R_{12}}\left\{\sum_{j\in s_{i}}f_{ij}\right\}\prod_{i=R_{1}+\cdots +R_{12}+1}^{R_{1}+\cdots +R_{13}}\left\{\sum_{j\in s_{i}}f_{ij}\right\}\\ \;\times \prod_{i=R_{1}+\cdots +R_{13}+1}^{R_{1}+\cdots +R_{14}}\left\{\sum_{j\in s_{i}}f_{ij}\right\}\prod_{i=R_{1}+\cdots +R_{14}+1}^{n_{u}}\left\{\sum_{j\in s_{i}}f_{ij}\right\}\times \prod_{i=1}^{n_{u}}\left\{{\left[{\bar{F}}_{1}\left(t_{i}\right)\right]}^{R_{i}}\right\}\\ \;\times \prod_{i=n_{u}+1}^{n_{u}+R_{15}}\left\{\sum_{j\in s_{i}}f_{ij}\right\}\prod_{i=n_{u}+R_{15}+1}^{n_{u}+R_{15}+R_{16}}\left\{\sum_{j\in s_{i}}f_{ij}\right\}\prod_{i=n_{u}+R_{15}+R_{16}+1}^{n_{u}+R_{15}+\cdots +R_{17}}\left\{\sum_{j\in s_{i}}f_{ij}\right\}\prod_{i=n_{u}+R_{15}+\cdots +R_{17}+1}^{n_{u}+R_{15}+\cdots +R_{18}}\left\{\sum_{j\in s_{i}}f_{ij}\right\}\prod_{i=n_{u}+R_{15}+\cdots +R_{18}+1}^{n_{u}+R_{15}+\cdots +R_{19}}\left\{\sum_{j\in s_{i}}f_{ij}\right\}\\ \;\times \prod_{i=n_{u}+R_{15}+\cdots +R_{19}+1}^{n_{u}+R_{15}+\cdots +R_{20}}\left\{\sum_{j\in s_{i}}f_{ij}\right\}\prod_{i=n_{u}+R_{15}+\cdots +R_{20}+1}^{n_{u}+R_{15}+\cdots +R_{21}}\left\{\sum_{j\in s_{i}}f_{ij}\right\}\prod_{i=n_{u}+R_{15}+\cdots +R_{21}+1}^{n_{u}+R_{15}+\cdots +R_{22}}\left\{\sum_{j\in s_{i}}f_{ij}\right\}\prod_{i=n_{u}+R_{15}+\cdots +R_{22}+1}^{n_{u}+R_{15}+\cdots +R_{23}}\left\{\sum_{j\in s_{i}}f_{ij}\right\}\\ \;\times \prod_{i=n_{u}+R_{15}+\cdots +R_{23}+1}^{n_{u}+R_{15}+\cdots +R_{24}}\left\{\sum_{j\in s_{i}}f_{ij}\right\}\prod_{i=n_{u}+R_{15}+\cdots +R_{24}+1}^{n_{u}+R_{15}+\cdots +R_{25}}\left\{\sum_{j\in s_{i}}f_{ij}\right\}\prod_{i=n_{u}+R_{15}+\cdots +R_{25}+1}^{n_{u}+R_{15}+\cdots +R_{26}}\left\{\sum_{j\in s_{i}}f_{ij}\right\}\prod_{i=n_{u}+R_{15}+\cdots +R_{26}+1}^{n_{u}+R_{15}+\cdots +R_{27}}\left\{\sum_{j\in s_{i}}f_{ij}\right\}\\ \;\times \prod_{i=n_{u}+R_{15}+\cdots +R_{27}+1}^{n_{u}+R_{15}+\cdots +R_{28}}\left\{\sum_{j\in s_{i}}f_{ij}\right\}\prod_{i=n_{u}+R_{15}+\cdots +R_{28}+1}^{R}\left\{\sum_{j\in s_{i}}f_{ij}\right\}\times \prod_{i=n_{u}+1}^{R}\left\{{\left[{\bar{F}}_{2}\left(t_{i}\right)\right]}^{R_{i}}\right\}\times {\left[{\bar{F}}_{2}\left(\eta \right)\right]}^{R^{*}}\\ \;\propto \prod_{i=1}^{n_{u}}\left\{\lambda e^{t_{i}}\exp\left(-2\lambda \left(e^{t_{i}}-1\right)\right)\left[1-\exp\left(-2\lambda \left(e^{t_{i}}-1\right)\right)\right]\right\}\times \prod_{i=1}^{n_{u}}{\left\{\exp\left(-2\lambda \left(e^{t_{i}}-1\right)\right)\left[2-\exp\left(-2\lambda \left(e^{t_{i}}-1\right)\right)\right]\right\}}^{R_{i}}\\ \;\times \prod_{i=n_{u}+1}^{R}\left\{\alpha \lambda e^{\left(\tau +\alpha (t_{i}-\tau )\right)}\exp\left(-2\lambda \left(e^{\left(\tau +\alpha (t_{i}-\tau )\right)}-1\right)\right)\left[1-\exp\left(-2\lambda \left(e^{\left(\tau +\alpha (t_{i}-\tau )\right)}-1\right)\right)\right]\right\}\\ \;\times \prod_{i=n_{u}+1}^{R}{\left\{\exp\left(-2\lambda \left(e^{\left(\tau +\alpha (t_{i}-\tau )\right)}-1\right)\right)\left[2-\exp\left(-2\lambda \left(e^{\left(\tau +\alpha (t_{i}-\tau )\right)}-1\right)\right)\right]\right\}}^{R_{i}}\\ \;\times {\left\{\exp\left(-2\lambda \left(e^{\left(\tau +\alpha (\eta -\tau )\right)}-1\right)\right)\left[2-\exp\left(-2\lambda \left(e^{\left(\tau +\alpha (\eta -\tau )\right)}-1\right)\right)\right]\right\}}^{R^{*}}. \end{array}$$ (9)

The log-likelihood function *l* = ln*L*(*λ*,*a*) can be written as

$$\begin{array}{l} l\propto R\ln\lambda +n_{v}\ln\alpha +\sum_{i=1}^{n_{u}}\left[t_{i}-2\lambda A\left(t_{i}\right)+\ln\left(1-W^{-1}\left(t_{i}\right)\right)\right]+\sum_{i=1}^{n_{u}}R_{i}\left[-2\lambda A\left(t_{i}\right)+\ln\left(2-W^{-1}\left(t_{i}\right)\right)\right]\\ \;+\sum_{i=n_{u}+1}^{R}\left[\tau +\alpha (t_{i}-\tau )-2\lambda B\left(t_{i}\right)+\ln\left(1-Q^{-1}\left(t_{i}\right)\right)\right]\\ \;+\sum_{i=n_{u}+1}^{R}R_{i}\left[-2\lambda B\left(t_{i}\right)+\ln\left(2-Q^{-1}\left(t_{i}\right)\right)\right]+R^{*}\left[-2\lambda B\left(\eta \right)+\ln\left(2-Q^{-1}\left(\eta \right)\right)\right], \end{array}$$ (10)

where $A\left(t_{i}\right)=e^{t_{i}}-1$, $W^{-1}\left(t_{i}\right)=1/W\left(t_{i}\right)=\exp\left(-2\lambda \left(e^{t_{i}}-1\right)\right)$, $B\left(t_{i}\right)=e^{{}^{\left(\tau +\alpha (t_{i}-\tau )\right)}}-1$, and $Q^{-1}\left(t_{i}\right)=1/Q\left(t_{i}\right)=\exp\left(-2\lambda \left(e^{{}^{\left(\tau +\alpha (t_{i}-\tau )\right)}}-1\right)\right)$.

Setting the partial derivatives of *l* with respect to *λ* and *α* to zero, we can obtain

$$\begin{array}{l} \frac{\partial l}{\partial \lambda }=\frac{R}{\lambda }+\sum_{i=1}^{n_{u}}2A\left(t_{i}\right)\left[\frac{1}{W\left(t_{i}\right)-1}-1\right]+\sum_{i=1}^{n_{u}}2R_{i}A\left(t_{i}\right)\left[\frac{1}{2W\left(t_{i}\right)-1}-1\right]\\ \;+\sum_{i=n_{u}+1}^{R}2B\left(t_{i}\right)\left[\frac{1}{Q\left(t_{i}\right)-1}-1\right]+\sum_{i=n_{u}+1}^{R}2R_{i}B\left(t_{i}\right)\left[\frac{1}{2Q\left(t_{i}\right)-1}-1\right]+2R^{*}B\left(\eta \right)\left[\frac{1}{2Q\left(\eta \right)-1}-1\right]=0 \end{array}$$

$$\begin{array}{l} \frac{\partial l}{\partial \alpha }=\frac{n_{v}}{\alpha }+\sum_{i=n_{u}+1}^{R}\left(t_{i}-\tau \right)\left[1-2\lambda \left(B\left(t_{i}\right)+1\right)+\frac{2\lambda \left(B\left(t_{i}\right)+1\right)}{Q\left(t_{i}\right)-1}\right]\\ \;+\sum_{i=n_{u}+1}^{R}2\lambda R_{i}\left(t_{i}-\tau \right)\left(B\left(t_{i}\right)+1\right)\left(\frac{1}{2Q\left(t_{i}\right)-1}-1\right)+2\lambda R^{*}\left(\eta -\tau \right)\left(B\left(\eta \right)+1\right)\left(\frac{1}{2Q\left(\eta \right)-1}-1\right)=0 \end{array}$$

Solve the above equations by using an iterative method such as Newton-Raphson, we can derive the MLEs of *λ* and *α*.

Similar to the hybrid systems (a), we can get the likelihood function for the hybrid systems (b).

$$\begin{array}{l} L\left(\lambda ,a\right)\propto \prod_{i=1}^{n_{u}}\left\{\left(\sum_{j\in s_{i}}f_{ij}\right)\cdot {\left[{\bar{F}}_{1}\left(t_{i}\right)\right]}^{R_{i}}\right\}\prod_{i=n_{u}+1}^{R}\left\{\left(\sum_{j\in s_{i}}f_{ij}\right)\cdot {\left[{\bar{F}}_{2}\left(t_{i}\right)\right]}^{R_{i}}\right\}\cdot {\left[{\bar{F}}_{2}\left(\eta \right)\right]}^{R^{*}}\\ \;\propto \prod_{i=1}^{n_{u}}\left\{\lambda e^{t_{i}}\exp\left(-2\lambda \left(e^{t_{i}}-1\right)\right)\left[1-\exp\left(-\lambda \left(e^{t_{i}}-1\right)\right)\right]\left[2-\exp\left(-\lambda \left(e^{t_{i}}-1\right)\right)\right]\right\}\\ \;\times \prod_{i=1}^{n_{u}}{\left\{\exp\left(-2\lambda \left(e^{t_{i}}-1\right)\right){\left[2-\exp\left(-\lambda \left(e^{t_{i}}-1\right)\right)\right]}^{2}\right\}}^{R_{i}}\\ \;\times \prod_{i=n_{u}+1}^{R}\left\{\alpha \lambda e^{\left(\tau +\alpha (t_{i}-\tau )\right)}\exp\left(-2\lambda \left(e^{\left(\tau +\alpha (t_{i}-\tau )\right)}-1\right)\right)\left[1-\exp\left(-\lambda \left(e^{\left(\tau +\alpha (t_{i}-\tau )\right)}-1\right)\right)\right]\left[2-\exp\left(-\lambda \left(e^{\left(\tau +\alpha (t_{i}-\tau )\right)}-1\right)\right)\right]\right\}\\ \;\times \prod_{i=n_{u}+1}^{R}{\left\{\exp\left(-2\lambda \left(e^{\left(\tau +\alpha (t_{i}-\tau )\right)}-1\right)\right){\left[2-\exp\left(-\lambda \left(e^{\left(\tau +\alpha (t_{i}-\tau )\right)}-1\right)\right)\right]}^{2}\right\}}^{R_{i}}\\ \;\times {\left\{\exp\left(-2\lambda \left(e^{\left(\tau +\alpha (\eta -\tau )\right)}-1\right)\right){\left[2-\exp\left(-\lambda \left(e^{\left(\tau +\alpha (\eta -\tau )\right)}-1\right)\right)\right]}^{2}\right\}}^{R^{*}}. \end{array}$$ (11)

From Eq.(11),the log-likelihood function *l* = ln*L*(*λ*,*a*) is given by

$$\begin{array}{l} l\propto R\ln\lambda +n_{v}\ln\alpha +\sum_{i=1}^{n_{u}}\left[t_{i}-2\lambda A\left(t_{i}\right)+\ln\left(1-C^{-1}\left(t_{i}\right)\right)+\ln\left(2-C^{-1}\left(t_{i}\right)\right)\right]+\sum_{i=1}^{n_{u}}R_{i}\left[-2\lambda A\left(t_{i}\right)+2\ln\left(2-C^{-1}\left(t_{i}\right)\right)\right]\\ \;+\sum_{i=n_{u}+1}^{R}\left[\tau +\alpha (t_{i}-\tau )-2\lambda B\left(t_{i}\right)+\ln\left(1-D^{-1}\left(t_{i}\right)\right)+\ln\left(2-D^{-1}\left(t_{i}\right)\right)\right]\\ \;+\sum_{i=n_{u}+1}^{R}R_{i}\left[-2\lambda B\left(t_{i}\right)+2\ln\left(2-D^{-1}\left(t_{i}\right)\right)\right]+R^{*}\left[-2\lambda B\left(\eta \right)+\ln\left(2-D^{-1}\left(\eta \right)\right)\right], \end{array}$$ (12)

where $A\left(t_{i}\right)=e^{t_{i}}-1$, $C^{-1}\left(t_{i}\right)=1/C\left(t_{i}\right)=\exp\left(-\lambda \left(e^{t_{i}}-1\right)\right)$, $B\left(t_{i}\right)=e^{{}^{\left(\tau +\alpha (t_{i}-\tau )\right)}}-1$, and $D^{-1}\left(t_{i}\right)=1/D\left(t_{i}\right)=\exp\left(-\lambda \left(e^{{}^{\left(\tau +\alpha (t_{i}-\tau )\right)}}-1\right)\right)$.

From Eq (12), we obtain the partial derivatives of *l* with respect to *λ* and *α* and equating them to zero as follows

$$\begin{array}{l} \frac{\partial l}{\partial \lambda }=\frac{R}{\lambda }+\sum_{i=1}^{n_{u}}A\left(t_{i}\right)\left[\frac{1}{C\left(t_{i}\right)-1}+\frac{1}{2C\left(t_{i}\right)-1}-2\right]+\sum_{i=1}^{n_{u}}2R_{i}A\left(t_{i}\right)\left[\frac{1}{2C\left(t_{i}\right)-1}-1\right]\\ \;+\sum_{i=n_{u}+1}^{R}B\left(t_{i}\right)\left[\frac{1}{D\left(t_{i}\right)-1}+\frac{1}{2D\left(t_{i}\right)-1}-2\right]+\sum_{i=n_{u}+1}^{R}2R_{i}B\left(t_{i}\right)\left[\frac{1}{2D\left(t_{i}\right)-1}-1\right]+2R^{*}B\left(\eta \right)\left[\frac{1}{2D\left(\eta \right)-1}-1\right]=0 \end{array}$$

$$\begin{array}{l} \frac{\partial l}{\partial \alpha }=\frac{n_{v}}{\alpha }+\sum_{i=n_{u}+1}^{R}\left(t_{i}-\tau \right)\left[1-2\lambda \left(B\left(t_{i}\right)+1\right)+\frac{\lambda \left(B\left(t_{i}\right)+1\right)}{D\left(t_{i}\right)-1}+\frac{\lambda \left(B\left(t_{i}\right)+1\right)}{2D\left(t_{i}\right)-1}\right]\\ \;+\sum_{i=n_{u}+1}^{R}2\lambda R_{i}\left(t_{i}-\tau \right)\left(B\left(t_{i}\right)+1\right)\left(\frac{1}{2D\left(t_{i}\right)-1}-1\right)+2\lambda R^{*}\left(\eta -\tau \right)\left(B\left(\eta \right)+1\right)\left(\frac{1}{2D\left(\eta \right)-1}-1\right)=0 \end{array}$$

The above equations can be solved by using an iterative method such as Newton-Raphson, we can derive the MLEs of parameter *λ* and *α*.

According to the invariance of maximum likelihood estimate, we can easily derive MLE of survival function of system at use stress level, it is denoted by ${\hat{\bar{F}}}_{1}\left(t\right)$.

For the hybrid systems (a),

$${\hat{\bar{F}}}_{1}\left(t\right)=\exp\left(-2\hat{\lambda }\left(e^{t}-1\right)\right)\left[2-\exp\left(-2\hat{\lambda }\left(e^{t}-1\right)\right)\right],\;t>0,$$

For the hybrid systems (b),

$${\hat{\bar{F}}}_{1}\left(t\right)=\exp\left(-2\hat{\lambda }\left(e^{t}-1\right)\right){\left\{\left[2-\exp\left(-\hat{\lambda }\left(e^{t}-1\right)\right)\right]\right\}}^{2},\;t>0,$$

where $\hat{\lambda }$ is the MLE of parameter *λ*.

## Confidence interval

### Approximate confidence interval

The observed Fisher information matrix is given by

$$F=-{\left(\frac{\partial^{2}\hat{l}}{\partial \phi_{p}\partial \phi_{q}}\right)}_{2\times 2}=-{\left[\begin{array}{cc} \frac{\partial^{2}l}{\partial \lambda^{2}} & \frac{\partial^{2}l}{\partial \lambda \partial \alpha }\\ \frac{\partial^{2}l}{\partial \alpha \partial \lambda } & \frac{\partial^{2}l}{\partial \alpha^{2}} \end{array}\right]}_{↓(\hat{\lambda },\hat{\alpha })},p,q=1,2;\phi_{1}=\lambda ,\phi_{2}=\alpha$$

where *F*^−1^ is the asymptotic variance-covariance matrix for $\hat{\lambda }$ and $\hat{\alpha }$.

The approximate 1001-*γ*% confidence intervals for the parameters *λ* and *α* can be presented by

$$(\hat{\lambda }-z_{\gamma /2}\sqrt{F_{11}^{-1}},\hat{\lambda }+z_{\gamma /2}\sqrt{F_{11}^{-1}}),\;(\hat{\alpha }-z_{\gamma /2}\sqrt{F_{22}^{-1}},\hat{\alpha }+z_{\gamma /2}\sqrt{F_{22}^{-1}}),$$

where *z*_*γ*/2_ is the upper(*γ*/2) percentile of the standard normal distribution.

For the hybrid systems (a), we get the second partial derivatives of *l* with respect to *λ* and *α*.

$$\begin{array}{l} \frac{\partial^{2}l}{\partial \lambda^{2}}=-\frac{R}{\lambda^{2}}+\sum_{i=1}^{n_{u}}\left\{\frac{-2A(t_{i})}{{\left(W(t_{i})-1\right)}^{2}}\frac{\partial W(t_{i})}{\partial \lambda }+\frac{-4R_{i}A(t_{i})}{{\left(2W(t_{i})-1\right)}^{2}}\frac{\partial W(t_{i})}{\partial \lambda }\right\}\\ \;+\sum_{i=n_{u}+1}^{R}\left\{\frac{-2B(t_{i})}{{\left(Q(t_{i})-1\right)}^{2}}\frac{\partial Q(t_{i})}{\partial \lambda }+\frac{-4R_{i}B(t_{i})}{{\left(2Q(t_{i})-1\right)}^{2}}\frac{\partial Q(t_{i})}{\partial \lambda }\right\}+R^{*}\left[\frac{-4B(\eta )}{{\left(2Q(\eta )-1\right)}^{2}}\frac{\partial Q(\eta )}{\partial \lambda }\right], \end{array}$$

$$\begin{array}{l} \frac{\partial^{2}l}{\partial \alpha \partial \lambda }=\sum_{i=n_{u}+1}^{R}2\left(t_{i}-\tau \right)\left(B\left(t_{i}\right)+1\right)\left\{\frac{1}{Q(t_{i})-1}-\frac{\lambda }{{\left(Q(t_{i})-1\right)}^{2}}\cdot \frac{\partial Q(t_{i})}{\partial \lambda }-1\right\}\\ \;+\sum_{i=n_{u}+1}^{R}2R_{i}\left(t_{i}-\tau \right)\left(B\left(t_{i}\right)+1\right)\left\{\frac{1}{2Q(t_{i})-1}-\frac{2\lambda }{{\left(2Q(t_{i})-1\right)}^{2}}\cdot \frac{\partial Q(t_{i})}{\partial \lambda }-1\right\}\\ \;+2R^{*}\left(\eta -\tau \right)\left(B\left(\eta \right)+1\right)\left\{\frac{1}{2Q(\eta )-1}-\frac{2\lambda }{{\left(2Q(\eta )-1\right)}^{2}}\cdot \frac{\partial Q(\eta )}{\partial \lambda }-1\right\}, \end{array}$$

$$\begin{array}{l} \frac{\partial^{2}l}{\partial \alpha^{2}}=-\frac{n_{v}}{\alpha^{2}}+\sum_{i=n_{u}+1}^{R}2\lambda \left(t_{i}-\tau \right)\left\{\frac{1}{Q(t_{i})-1}\frac{\partial B(t_{i})}{\partial \alpha }-\frac{B(t_{i})+1}{{\left(Q(t_{i})-1\right)}^{2}}\frac{\partial Q(t_{i})}{\partial \alpha }-\frac{\partial B(t_{i})}{\partial \alpha }\right\}\\ \;+\sum_{i=n_{u}+1}^{R}2\lambda R_{i}\left(t_{i}-\tau \right)\left\{\frac{1}{2Q(t_{i})-1}\frac{\partial B(t_{i})}{\partial \alpha }-\frac{2(B(t_{i})+1)}{{\left(2Q(t_{i})-1\right)}^{2}}\frac{\partial Q(t_{i})}{\partial \alpha }-\frac{\partial B(t_{i})}{\partial \alpha }\right\}\\ \;+2\lambda R^{*}\left(\eta -\tau \right)\left\{\frac{1}{2Q(\eta )-1}\frac{\partial B(\eta )}{\partial \alpha }-\frac{2(B(\eta )+1)}{{\left(2Q(\eta )-1\right)}^{2}}\frac{\partial Q(\eta )}{\partial \alpha }-\frac{\partial B(\eta )}{\partial \alpha }\right\}, \end{array}$$

where $\frac{\partial B(t_{i})}{\partial \alpha }=\left(t_{i}-\tau \right)\left(B\left(t_{i}\right)+1\right)$, $\frac{\partial W(t_{i})}{\partial \lambda }=2A(t_{i})W(t_{i})$, $\frac{\partial Q(t_{i})}{\partial \lambda }=2B\left(t_{i}\right)Q(t_{i})$, and $\frac{\partial Q(t_{i})}{\partial \alpha }=2\lambda \left(t_{i}-\tau \right)\left(B\left(t_{i}\right)+1\right)Q(t_{i})$.

Similarly, for the hybrid systems (b).

$$\begin{array}{l} \frac{\partial^{2}l}{\partial \lambda^{2}}=-\frac{R}{\lambda^{2}}+\sum_{i=1}^{n_{u}}\left\{\frac{-A(t_{i})}{{\left(C(t_{i})-1\right)}^{2}}\frac{\partial C(t_{i})}{\partial \lambda }+\frac{-2A(t_{i})}{{\left(2C(t_{i})-1\right)}^{2}}\frac{\partial C(t_{i})}{\partial \lambda }+\frac{-4R_{i}A(t_{i})}{{\left(2C(t_{i})-1\right)}^{2}}\frac{\partial C(t_{i})}{\partial \lambda }\right\}\\ \;+\sum_{i=n_{u}+1}^{R}\left\{\frac{-B(t_{i})}{{\left(D(t_{i})-1\right)}^{2}}\frac{\partial D(t_{i})}{\partial \lambda }+\frac{-2B(t_{i})}{{\left(2D(t_{i})-1\right)}^{2}}\frac{\partial D(t_{i})}{\partial \lambda }+\frac{-4R_{i}B(t_{i})}{{\left(2D(t_{i})-1\right)}^{2}}\frac{\partial D(t_{i})}{\partial \lambda }\right\}+R^{*}\left[\frac{-4B(\eta )}{{\left(2D(\eta )-1\right)}^{2}}\frac{\partial D(\eta )}{\partial \lambda }\right], \end{array}$$

$$\begin{array}{l} \frac{\partial^{2}l}{\partial \alpha \partial \lambda }=\sum_{i=n_{u}+1}^{R}\left(t_{i}-\tau \right)\left(B\left(t_{i}\right)+1\right)\left\{\frac{1}{D(t_{i})-1}-\frac{\lambda }{{\left(D(t_{i})-1\right)}^{2}}\cdot \frac{\partial D(t_{i})}{\partial \lambda }+\frac{1}{2D(t_{i})-1}-\frac{2\lambda }{{\left(2D(t_{i})-1\right)}^{2}}\cdot \frac{\partial D(t_{i})}{\partial \lambda }-2\right\}\\ \;+\sum_{i=n_{u}+1}^{R}2R_{i}\left(t_{i}-\tau \right)\left(B\left(t_{i}\right)+1\right)\left\{\frac{1}{2D(t_{i})-1}-\frac{2\lambda }{{\left(2D(t_{i})-1\right)}^{2}}\cdot \frac{\partial D(t_{i})}{\partial \lambda }-1\right\}\\ \;+2R^{*}\left(\eta -\tau \right)\left(B\left(\eta \right)+1\right)\left\{\frac{1}{2D(\eta )-1}-\frac{2\lambda }{{\left(2D(\eta )-1\right)}^{2}}\cdot \frac{\partial D(\eta )}{\partial \lambda }-1\right\}, \end{array}$$

$$\begin{array}{l} \frac{\partial^{2}l}{\partial \alpha^{2}}=-\frac{n_{v}}{\alpha^{2}}+\sum_{i=n_{u}+1}^{R}\lambda \left(t_{i}-\tau \right)\left\{\frac{1}{D(t_{i})-1}\frac{\partial B(t_{i})}{\partial \alpha }-\frac{B(t_{i})+1}{{\left(D(t_{i})-1\right)}^{2}}\frac{\partial D(t_{i})}{\partial \alpha }+\frac{1}{2D(t_{i})-1}\frac{\partial B(t_{i})}{\partial \alpha }-\frac{2\left(B(t_{i})+1\right)}{{\left(2D(t_{i})-1\right)}^{2}}\frac{\partial D(t_{i})}{\partial \alpha }-2\cdot \frac{\partial B(t_{i})}{\partial \alpha }\right\}\\ \;+\sum_{i=n_{u}+1}^{R}2\lambda R_{i}\left(t_{i}-\tau \right)\left\{\frac{1}{2D(t_{i})-1}\frac{\partial B(t_{i})}{\partial \alpha }-\frac{2(B(t_{i})+1)}{{\left(2D(t_{i})-1\right)}^{2}}\frac{\partial D(t_{i})}{\partial \alpha }-\frac{\partial B(t_{i})}{\partial \alpha }\right\}\\ \;+2\lambda R^{*}\left(\eta -\tau \right)\left\{\frac{1}{2D(\eta )-1}\frac{\partial B(\eta )}{\partial \alpha }-\frac{2(B(\eta )+1)}{{\left(2D(\eta )-1\right)}^{2}}\frac{\partial D(\eta )}{\partial \alpha }-\frac{\partial B(\eta )}{\partial \alpha }\right\}, \end{array}$$

where $\frac{\partial B(t_{i})}{\partial \alpha }=\left(t_{i}-\tau \right)\left(B\left(t_{i}\right)+1\right)$, $\frac{\partial C(t_{i})}{\partial \lambda }=A(t_{i})C(t_{i})$, $\frac{\partial D(t_{i})}{\partial \lambda }=B\left(t_{i}\right)D(t_{i})$, $\frac{\partial Q(t_{i})}{\partial \alpha }=\lambda \left(t_{i}-\tau \right)\left(B\left(t_{i}\right)+1\right)D(t_{i})$.

### Bootstrap confidence intervals

The bootstrap is a resampling method for statistical inference. In this section, we use the parametric bootstrap method to construct Studentized-t (Stud-t) bootstrap confidence interval (CI) for the unknown parameters *λ* and *a*, which was suggested by Hall [30] as follows.

1. Based on type-II progressively hybrid censored sample and masked data, we can obtain the MLE of *ψ*, $\hat{\psi }=\left({\hat{\varphi }}_{1},{\hat{\varphi }}_{2}\right)$ (where ${\hat{\varphi }}_{1}=\hat{\lambda }$, ${\hat{\varphi }}_{2}=\hat{a}$).
2. Based on the Prefix the values of *n*,*m*,*τ*,*η* along with the same *R*_1_,*R*_2_⋯,*R*_*m*_, Generated type-II progressively hybrid censored bootstrap samples and compute the bootstrap estimate ${\hat{\varphi }}_{1}^{*}$ and ${\hat{\varphi }}_{2}^{*}$ of the parameter by the method proposed in Section 3 (where ${\hat{\varphi }}_{1}^{*}={\hat{\lambda }}^{*}$, ${\hat{\varphi }}_{2}^{*}={\hat{a}}^{*}$) as with (1).
3. Repeat steps (2) N^*^ times representing N^*^ different bootstrap samples. The value of N^*^ has been taken to be 2000.
4. Arrange all ${\hat{\psi }}^{*}=\left({\hat{\varphi }}_{1}^{*},{\hat{\varphi }}_{2}^{*}\right)$ in an ascending order to derive the bootstrap sample $\left({\hat{\varphi }}_{\delta }^{*[1]},{\hat{\varphi }}_{\delta }^{*[2]}\ldots ,{\hat{\varphi }}_{\delta }^{*[N^{*}]}\right),\delta =1,2$.

The Stud-t bootstrap CIs can be given as follows. Firstly, find the order statistics $\sigma_{\delta }^{*[1]}<\sigma_{\delta }^{*[2]}<\ldots <\sigma_{\delta }^{*[N^{*}]},\delta =1,2$, where $\sigma_{\delta }^{*[d]}=\frac{{\hat{\varphi }}_{\delta }^{*[d]}-{\hat{\varphi }}_{\delta }}{\sqrt{\mathrm{var}\left({\hat{\varphi }}_{\delta }^{*[d]}\right)}},d=1,2,\ldots ,N^{*},\delta =1,2.$

Secondly, choose the interval $\left(\sigma_{\delta L}^{*},\sigma_{\delta U}^{*}\right)$ from all possible 1001-*γ*% CIs of the form $\left(\sigma_{\delta }^{*[h]},\sigma_{\delta }^{*[1-\gamma N^{*}+h]}\right),h=1,2,\ldots$, γN^*^, *δ* = 1,2 when the width of CI is a minimum.

Finally, the two-sided Stud-t BCI for *φ*_*δ*_ can be given by $\left({\hat{\varphi }}_{\delta }-\sigma_{\delta U}^{*}\sqrt{\mathrm{var}\left({\hat{\varphi }}_{\delta }\right)},{\hat{\varphi }}_{\delta }-\sigma_{\delta L}^{*}\sqrt{\mathrm{var}\left({\hat{\varphi }}_{\delta }\right)}\right)$.

## Simulation studies

In this section, we consider only the simulation studies for the hybrid system (a), and the hybrid system (b) can be similarly discussed. Monte Carlo simulation studies are conducted to discuss the performance of the MLEs in terms of their mean square errors (MSEs) for different choices of *n*, *m*, *τ* and *η* values based on masked hybrid system lifetime data in step-stress partially accelerated life test with Type-II PHC. Also, 95% approximate confidence intervals (ACIs) and Stud-t bootstrap confidence intervals (BCIs) of the model parameters are constructed and their lengths are computed and presented with associated coverage probabilities (CP).

Three different progressive censoring schemes are considered in the simulation study:

CS 1: *R*_1_ = ⋯ = *R*_*m*−1_ = 0,*R*_*m*_ = *n*−*m*,
CS 2: *R*_1_ = *n*−*m*, *R*_2_ = ⋯ = *R*_*m*_ = 0,
CS 3: *R*_1_ = *R*_2_ = ⋯ = *R*_*m*−11_ = 0, *R*_*m*−10_ = ⋯ = *R*_*m*−1_ = 1, *R*_*m*_ = *n*−*m*−10.

The simulation studies are carried out according to the following algorithm:

1. Specify the values of *n*,*m*,*τ*,*η*, *R*_1_,*R*_2_⋯,*R*_*m*_ and the values of parameters *λ*, *α*.
2. Generate a random sample with size n from the random variable T given by Eq (5) and sort it. The random variable with PDF in Eq (2) can be easily generated. For example, if *U* represents a uniform random variable from [0, 1], then *T* = ln[1−[ln(1−*U*)/*λ*]] has the PDF given by Eq (2) if *t* ≤ *τ*. But if *t* > *τ* then *T* = *τ* + [ln(1−(ln(1−*U*)/*λ*)−*τ*)]/*α* has the PDF given by Eq (6).
3. For given the values of *n*,*m*,*τ*,*η*(*η*>*τ*), *R*_1_,*R*_2_⋯,*R*_*m*_ and the values of parameters *λ*, *α*, the Type-II progressively hybrid censored sample is generated.
4. Based on the Type-II progressive hybrid censored sample, generated the Type-II progressively hybrid censored sample with masked data:
  Case Ⅰ: $\left\{(t_{1},s_{1}),(t_{2},s_{2}),\cdots ,(t_{n_{u}},s_{n_{u}}),\cdots ,(t_{m},s_{m})\right\}$, if *τ* < *t*_*m*_ ≤ *η*,
  Case Ⅱ: $\left\{(t_{1},s_{1}),(t_{2},s_{2}),\cdots ,(t_{n_{u}},s_{n_{u}}),\cdots ,(t_{n_{u}+n_{v}},s_{n_{u}+n_{v}})\right\}$, if *t*_*m*_ > *η*.
5. Use the values of Type-II progressively hybrid censored sample with masked data to compute the MLEs, ACI and BCI of the model parameters using the method proposed in the section 4,
6. Replicate steps 2–5 2000 times, and compute the average values of MLEs of the parameters as well as associated MSEs.
7. Compute the average values of intervals lengths (ILs) as well as the associated coverage probabilities with each parameter using confidence level 0.95.
8. Steps 1–7 are done with different values of *n*,*m*,*τ*,*η*(*η*>*τ*), *R*_1_,*R*_2_⋯,*R*_*m*_, *λ* and *α*.

Tables 1 and 2 present the average of MLE (A-MLE) and corresponding mean square error (MSE) of the parameter *λ* and the acceleration factor *α* for the different values of *n*,*m*,*τ*,*η* and the censoring schemes (CS), while the 95% CIs, corresponding interval length (IL) and CP of the parameter *λ* and *a* are given in Tables 3 and 4.

**Table 1:** A-MLE for parameters and MSE (*λ* = 1.2, *α* = 1.5 and *η* = 0.7).

A-MLE ($\hat{\lambda }$)				A-MLE ($\hat{a}$)		MSE ($\hat{\lambda }$)		MSE ($\hat{a}$)	
(*n*,*m*)	CS	*τ* = 0.1	*τ* = 0.3	*τ* = 0.1	*τ* = 0.3	*τ* = 0.1	*τ* = 0.3	*τ* = 0.1	*τ* = 0.3
(80,40)	1	1.2934	1.3401	1.1687	1.1265	0.0382	0.0421	0.1443	0.1567
	2	1.2881	1.0988	1.1107	1.6089	0.0235	0.0332	0.1356	0.1453
	3	1.1314	1.0887	1.2078	1.1898	0.0343	0.0375	0.1189	0.1124
(80,60)	1	1.2765	1.3101	1.2567	1.2065	0.0287	0.0239	0.1123	0.1209
	2	1.2909	1.1011	1.2102	1.1989	0.0212	0.0304	0.1078	0.1243
	3	1.1502	1.0912	1.2342	1.2001	0.0334	0.0365	0.0987	0.1098
(100,40)	1	1.2601	1.2824	1.1323	1.7298	0.0272	0.0254	0.1221	0.0868
	2	1.1408	1.3098	1.7001	1.2912	0.0213	0.0265	0.0675	0.0712
	3	1.1118	1.0765	1.2409	1.2328	0.0243	0.0253	0.0743	0.0674
(100,60)	1	1.1786	1.2754	1.2898	1.2398	0.0175	0.0189	0.0349	0.0432
	2	1.1456	1.2908	1.6487	1.2376	0.0214	0.0213	0.0498	0.0467
	3	1.2072	1.1468	1.2921	1.2754	0.0153	0.0208	0.0423	0.0556

**Table 2:** A-MLE for parameters and MSE (*λ* = 1.2, *α* = 1.5 and *η* = 0.7).

A-MLE ($\hat{\lambda }$)				A-MLE ($\hat{a}$)		MSE ($\hat{\lambda }$)		MSE ($\hat{a}$)	
(*n*,*m*)	CS	*τ* = 0.1	*τ* = 0.3	*τ* = 0.1	*τ* = 0.3	*τ* = 0.1	*τ* = 0.3	*τ* = 0.1	*τ* = 0.3
(80,40)	1	1.2787	1.3354	1.1701	1.1346	0.0367	0.0412	0.1401	0.1467
	2	1.2875	1.0916	1.1123	1.6023	0.0205	0.0312	0.1323	0.1408
	3	1.1138	1.0576	1.2012	1.1943	0.0298	0.0401	0.1112	0.1156
(80,60)	1	1.2697	1.3021	1.2711	1.2212	0.0256	0.0212	0.1108	0.1211
	2	1.2876	1.1078	1.2254	1.1989	0.0212	0.0298	0.1005	0.1008
	3	1.1432	1.0876	1.2087	1.1965	0.0312	0.0324	0.0881	0.1012
(100,40)	1	1.2547	1.2756	1.1954	1.1656	0.0254	0.0212	0.1108	0.1081
	2	1.1498	1.3032	1.6987	1.3082	0.0206	0.0256	0.0609	0.0761
	3	1.1328	1.0932	1.2543	1.2301	0.0231	0.0277	0.0701	0.0612
(100,60)	1	1.1775	1.2546	1.2989	1.2416	0.0167	0.0176	0.0308	0.0431
	2	1.1507	1.2897	1.6187	1.2409	0.0215	0.0219	0.0421	0.0474
	3	1.2054	1.1576	1.3001	1.2842	0.0131	0.0178	0.0386	0.0412

**Table 3:** 95% CI for parameters, IL and CP of CI (*λ* = 1.2, *a* = 1.5, *τ* = 0.3 and *η* = 0.7).

(*n*,*m*)	CS	Para.	ACI, IL, CP	BCI, IL, CP
(80,40)	1	*λ*	(0.8256,1.6523), 0.8267, (0.937)	(0.8563,1.5312), 0.6749, (0.944)
		*a*	(0.8897,1.9442), 1.0545, (0.942)	(1.0067,1.8787), 0.8720, (0.957)
	2	*λ*	(0.8312,1.4787), 0.6475, (0.941)	(0.8397,1.4396), 0.5999, (0.952)
		*a*	(1.0223,1.9009), 0.8786, (0.957)	(1.0212,1.8554), 0.8342, (0.942)
	3	*λ*	(0.8336,1.4698), 0.6362, (0.943)	(0.8521,1.4523), 0.6002, (0.933)
		*a*	(1.0543,1.9012), 0.8469, (0.945)	(1.0765,1.8432), 0.7667, (0.956)
(80,60)	1	*λ*	(0.8339,1.4476), 0.6137, (0.951)	(0.8401,1.4234), 0.5833, (0.949)
		*a*	(1.1121,1.9112), 0.7991, (0.939)	(1.1132,1.8245), 0.7113, (0.957)
	2	*λ*	(0.8412,1.4422), 0.6010, (0.959)	(0.8398,1.4506), 0.6108, (0.952)
		*a*	(1.0134,1.8156), 0.8022, (0.961)	(1.0145,1.8103), 0.7958, (0.947)
	3	*λ*	(0.8322,1.4276), 0.5954, (0.949)	(0.8564,1.4301), 0.5737, (0.958)
		*a*	(1.0127,1.8314), 0.8187, (0.946)	(1.1084,1.8123), 0.7039, (0.951)
(100,40)	1	*λ*	(0.8427,1.4378), 0.5951, (0.963)	(0.8423,1.4211), 0.5788, (0.967)
		*a*	(1.0122,1.8676), 0.8554, (0.945)	(1.1211,1.7653), 0.6442, (0.956)
	2	*λ*	(0.8312,1.4091), 0.5779, (0.954)	(0.8465,1.4089), 0.5624, (0.947)
		*a*	(1.1101,1.7531), 0.6430, (0.965)	(1.1379,1.6901), 0.5522, (0.963)
	3	*λ*	(0.8442,1.4308), 0.5866, (0.952)	(0.8698,1.4128), 0.5430, (0.971)
		*a*	(1.1281,1.7361), 0.6080, (0.947)	(1.1498,1.6213), 0.4715, (0.958)
(100,60)	1	*λ*	(0.8809,1.4218), 0.5409, (0.958)	(0.8876,1.4198), 0.5322, (0.951)
		*a*	(1.1265,1.8312), 0.7047, (0.946)	(1.1422,1.7041), 0.5619, (0.967)
	2	*λ*	(0.8678,1.4134), 0.5456, (0.952)	(0.8866,1.4112), 0.5246, (0.957)
		*a*	(1.1301,1.8221), 0.6920, (0.956)	(1.1531,1.7165), 0.5634, (0.961)
	3	*λ*	(0.8736,1.4087), 0.5351, (0.953)	(0.8809,1.4011), 0.5202, (0.952)
		*a*	(1.1408,1.7122), 0.5714, (0.961)	(1.1598,1.6987), 0.5389, (0.969)

**Table 4:** 95% CI for parameters, IL and CP of CI (*λ* = 1.2, *a* = 1.5, *τ* = 0.3 and *η* = 1.5).

(*n*,*m*)	CS	Para.	ACI, IL, CP	BCI, IL, CP
(80,40)	1	*λ*	(0.8277,1.6471), 0.8194, (0.941)	(0.8437,1.5124), 0.6687, (0.933)
		*a*	(0.8764,1.9341), 1.0577, (0.958)	(1.0981,1.8987), 0.8006, (0.947)
	2	*λ*	(0.8365,1.4456), 0.6091, (0.942)	(0.8278,1.4212), 0.5934, (0.967)
		*a*	(1.0134,1.9016), 0.8882, (0.957)	(1.0198,1.8679), 0.8481, (0.955)
	3	*λ*	(0.8445,1.4612), 0.6167, (0.952)	(0.8643,1.4501), 0.5858, (0.948)
		*a*	(0.9487,1.9001), 0.9514, (0.949)	(1.0675,1.8821), 0.8146, (0.961)
(80,60)	1	*λ*	(0.8398,1.4453), 0.6055, (0.945)	(0.8403,1.4212), 0.5809, (0.957)
		*a*	(1.0009,1.9101), 0.9092, (0.954)	(1.0112,1.8567), 0.8455, (0.949)
	2	*λ*	(0.8478,1.4409), 0.5931, (0.952)	(0.8422,1.4376), 0.5954, (0.955)
		*a*	(1.0113,1.8147), 0.8034, (0.967)	(1.1167,1.8133), 0.6966, (0.942)
	3	*λ*	(0.8378,1.4265), 0.5887, (0.948)	(0.8465,1.4267), 0.5802 (0.945)
		*a*	(1.0568,1.8228), 0.7660, (0.962)	(1.1371,1.8187), 0.6816, (0.953)
(100,40)	1	*λ*	(0.8456,1.4321), 0.5865, (0.957)	(0.8478,1.4278), 0.5800, (0.961)
		*a*	(1.1144,1.8765), 0.7621, (0.965)	(1.1231,1.7007), 0.5776, (0.959)
	2	*λ*	(0.8309,1.4087), 0.5778, (0.962)	(0.8412,1.4076), 0.5664, (0.964)
		*a*	(1.1012,1.7643), 0.6631, (0.939)	(1.1412,1.6885), 0.5473, (0.957)
	3	*λ*	(0.8436,1.4301), 0.5865, (0.961)	(0.8615,1.4238), 0.5623, (0.959)
		*a*	(1.0423,1.7445), 0.7022, (0.954)	(1.1035,1.6756), 0.5721, (0.971)
(100,60)	1	*λ*	(0.8813,1.4328), 0.5515, (0.955)	(0.8809,1.4167), 0.5358, (0.962)
		*a*	(1.1167,1.8008), 0.6841, (0.963)	(1.1577,1.7787), 0.6210, (0.965)
	2	*λ*	(0.8675,1.4212), 0.5537, (0.967)	(0.8712,1.4109), 0.5397, (0.968)
		*a*	(1.0271,1.8024), 0.7753, (0.962)	(1.1556,1.7298), 0.5742, (0.957)
	3	*λ*	(0.8721,1.4065), 0.5344, (0.952)	(0.8806,1.4002), 0.5196, (0.969)
		*a*	(1.1306,1.7117), 0.5811, (0.967)	(1.1501,1.6741), 0.5240, (0.971)

From Tables 1, 2, 3 and 4, it may be observed that

1. For fixed *η*,*τ* and *n*, the MSE of the MLE decreases as *m* increases, and A-MLE is closer to the real value. For fixed *η*, *τ* and *m*, the MSE of the MLE decreases as *n* increases.
2. For fixed *η*,*τ*, the MSE of the MLE decreases as *n* and *m* increase at the same time.
3. For fixed *n*,*m* and *η*, the MSE of the MLE decreases as *τ* decreases. For fixed *n*,*m* and *τ*, the MSE of the MLE decreases as *η* increases.
4. The bootstrap confidence interval has the more smaller length and more bigger coverage probabilities than the approximate confidence interval

Thus, the procedure proposed in this paper can achieve good estimation performance.

## Conclusions

In this paper, statistical analysis for two hybrid systems are studied based on masked data in SSPALT with Type-II progressive hybrid censoring. The maximum likelihood estimations for unknown parameters and acceleration factor are presented when the life time of system component follows exponential failure rate. In addition, approximate confidence interval and Stud-t bootstrap confidence interval of the model parameters are obtained by using the asymptotic distributions and parametric bootstrap method, respectively. The performance of estimation methods is assessed by simulation studies.

## Supporting information

S1 File The derivation of the simulation results in the Tables. The data of this paper are calculated by numerical simulation. All relevant data, including any instructions, equations, and parameters, needed to fully replicate the simulations described in the paper can be found within the paper or S1 File.
(PDF)
