# Supplementary material for: Statistical analysis for masked hybrid system lifetime data in step-stress partially accelerated life test with progressive hybrid censoring
Source: PLoS One. 2017 Oct 23;12(10):e0186417. doi: 10.1371/journal.pone.0186417 (PMC5653305; doi:10.1371/journal.pone.0186417)
Supplement: S1 File — The data of this paper are calculated by numerical simulation. All relevant data, including any instructions, equations, and parameters, needed to fully replicate the simulations described in the paper can be found within the paper or S1 File. (PDF) [file pone.0186417.s001.pdf]

The simulation studies are carried out according to the following algorithm:

(1) Specify the values of n, m, τ, η , $R_1, R_2 \cdots, R_m$ and the values of parameters λ , α .

(2) Generate a random sample with size n from the random variable T given by Eq. (5) and sort it. The random variable with PDF in Eq.(2) can be easily generated. For example, if U represents a uniform random variable from [0, 1], then $T = \ln[1-[\ln(1-U)/\lambda]]$ has the PDF given by Eq. (2) if $t \le \tau$. But if $t > \tau$ then $T = \tau + [\ln(1-(\ln(1-U)/\lambda)-\tau)]/\alpha$ has the PDF given by Eq. (6).

(3) For given the values of $n, m, \tau, \eta(\eta > \tau)$, $R_1, R_2 \cdots, R_m$ and the values of parameters λ , α , the Type-II progressively hybrid censored sample is generated.

(4) Based on the Type-II progressive hybrid censored sample, generated the Type-II progressively hybrid censored sample with masked data:

Case Ⅰ: $\{(t_1, s_1), (t_2, s_2), \cdots, (t_{n_u}, s_{n_u}), \cdots, (t_m, s_m)\}$, if $\tau < t_m \le \eta$,

Case Ⅱ: $\{(t_1, s_1), (t_2, s_2), \cdots, (t_{n_u}, s_{n_u}), \cdots, (t_{n_u+n_v}, s_{n_u+n_v})\}$, if $t_m > \eta$.

(5) Use the values of Type-II progressively hybrid censored sample with masked data to compute the MLEs, ACI and BCI of the model parameters using the method proposed in the section 4,

(6) Replicate steps 2-5 2000 times, and compute the average values of MLEs of the parameters as well as associated MSEs.

(7) Compute the average values of intervals lengths (ILs) as well as the associated coverage probabilities with each parameter using confidence level 0.95.

(8) Steps 1-7 are done with different values of $n, m, \tau, \eta(\eta > \tau)$, $R_1, R_2 \cdots, R_m$, λ and α .

Tables 1 and 2 present the average of MLE (A-MLE) and corresponding mean square error (MSE) of the parameter λ and the acceleration factor α for the different values of n, m, τ, η and the

censoring schemes (CS), while the 95% CIs, corresponding interval length (IL) and CP of the parameter λ and a are given in Tables 3 and 4.

Table 1. A-MLE for parameters and MSE (λ =1.2, α =1.5 and η=0.7)

		A-MLE ($\hat{\lambda}$)		A-MLE ($\hat{a}$)		MSE ($\hat{\lambda}$)		MSE ($\hat{a}$)	
(n,m)	CS	τ=0.1	τ=0.3	τ=0.1	τ=0.3	τ=0.1	τ=0.3	τ=0.1	τ=0.3
(80,40)	1	1.2934	1.3401	1.1687	1.1265	0.0382	0.0421	0.1443	0.1567
	2	1.2881	1.0988	1.1107	1.6089	0.0235	0.0332	0.1356	0.1453
	3	1.1314	1.0887	1.2078	1.1898	0.0343	0.0375	0.1189	0.1124
(80,60)	1	1.2765	1.3101	1.2567	1.2065	0.0287	0.0239	0.1123	0.1209
	2	1.2909	1.1011	1.2102	1.1989	0.0212	0.0304	0.1078	0.1243
	3	1.1502	1.0912	1.2342	1.2001	0.0334	0.0365	0.0987	0.1098
(100,40)	1	1.2601	1.2824	1.1323	1.7298	0.0272	0.0254	0.1221	0.0868
	2	1.1408	1.3098	1.7001	1.2912	0.0213	0.0265	0.0675	0.0712
	3	1.1118	1.0765	1.2409	1.2328	0.0243	0.0253	0.0743	0.0674
(100,60)	1	1.1786	1.2754	1.2898	1.2398	0.0175	0.0189	0.0349	0.0432
	2	1.1456	1.2908	1.6487	1.2376	0.0214	0.0213	0.0498	0.0467
	3	1.2072	1.1468	1.2921	1.2754	0.0153	0.0208	0.0423	0.0556

Table 2. A-MLE for parameters and MSE (λ =1.2, α =1.5 and η=0.7)

		A-MLE ($\hat{\lambda}$)		A-MLE ($\hat{a}$)		MSE ($\hat{\lambda}$)		MSE ($\hat{a}$)	
(n,m)	CS	τ=0.1	τ=0.3	τ=0.1	τ=0.3	τ=0.1	τ=0.3	τ=0.1	τ=0.3
(80,40)	1	1.2787	1.3354	1.1701	1.1346	0.0367	0.0412	0.1401	0.1467
	2	1.2875	1.0916	1.1123	1.6023	0.0205	0.0312	0.1323	0.1408
	3	1.1138	1.0576	1.2012	1.1943	0.0298	0.0401	0.1112	0.1156
(80,60)	1	1.2697	1.3021	1.2711	1.2212	0.0256	0.0212	0.1108	0.1211
	2	1.2876	1.1078	1.2254	1.1989	0.0212	0.0298	0.1005	0.1008
	3	1.1432	1.0876	1.2087	1.1965	0.0312	0.0324	0.0881	0.1012
(100,40)	1	1.2547	1.2756	1.1954	1.1656	0.0254	0.0212	0.1108	0.1081
	2	1.1498	1.3032	1.6987	1.3082	0.0206	0.0256	0.0609	0.0761
	3	1.1328	1.0932	1.2543	1.2301	0.0231	0.0277	0.0701	0.0612
(100,60)	1	1.1775	1.2546	1.2989	1.2416	0.0167	0.0176	0.0308	0.0431
	2	1.1507	1.2897	1.6187	1.2409	0.0215	0.0219	0.0421	0.0474
	3	1.2054	1.1576	1.3001	1.2842	0.0131	0.0178	0.0386	0.0412

Table 3. 95% CI for parameters, IL and CP of CI (λ =1.2, a =1.5, τ=0.3 and η=0.7)

(n,m)	CS	Para.	ACI , IL , CP	BCI, IL, CP
(80,40)	1	λ	(0.8256,1.6523), 0.8267, (0.937)	(0.8563,1.5312), 0.6749, (0.944)
		a	(0.8897,1.9442), 1.0545, (0.942)	(1.0067,1.8787), 0.8720, (0.957)
	2	λ	(0.8312,1.4787), 0.6475, (0.941)	(0.8397,1.4396), 0.5999, (0.952)
		a	(1.0223,1.9009), 0.8786 ,(0.957)	(1.0212,1.8554), 0.8342, (0.942)
	3	λ	(0.8336,1.4698), 0.6362, (0.943)	(0.8521,1.4523), 0.6002, (0.933)

		a	(1.0543,1.9012), 0.8469, (0.945)	(1.0765,1.8432), 0.7667, (0.956)
(80,60)	1	λ	(0.8339,1.4476), 0.6137, (0.951)	(0.8401,1.4234), 0.5833, (0.949)
		a	(1.1121,1.9112), 0.7991, (0.939)	(1.1132,1.8245), 0.7113, (0.957)
	2	λ	(0.8412,1.4422), 0.6010, (0.959)	(0.8398,1.4506), 0.6108, (0.952)
		a	(1.0134,1.8156), 0.8022, (0.961)	(1.0145,1.8103), 0.7958, (0.947)
	3	λ	(0.8322,1.4276), 0.5954, (0.949)	(0.8564,1.4301), 0.5737, (0.958)
		a	(1.0127,1.8314), 0.8187, (0.946)	(1.1084,1.8123), 0.7039, (0.951)
(100,40)	1	λ	(0.8427,1.4378), 0.5951, (0.963)	(0.8423,1.4211), 0.5788, (0.967)
		a	(1.0122,1.8676), 0.8554, (0.945)	(1.1211,1.7653), 0.6442, (0.956)
	2	λ	(0.8312,1.4091), 0.5779, (0.954)	(0.8465,1.4089), 0.5624, (0.947)
		a	(1.1101,1.7531), 0.6430, (0.965)	(1.1379,1.6901), 0.5522, (0.963)
	3	λ	(0.8442,1.4308), 0.5866, (0.952)	(0.8698,1.4128), 0.5430, (0.971)
		a	(1.1281,1.7361), 0.6080, (0.947)	(1.1498,1.6213), 0.4715, (0.958)
(100,60)	1	λ	(0.8809,1.4218), 0.5409, (0.958)	(0.8876,1.4198), 0.5322, (0.951)
		a	(1.1265,1.8312), 0.7047, (0.946)	(1.1422,1.7041), 0.5619, (0.967)
	2	λ	(0.8678,1.4134), 0.5456, (0.952)	(0.8866,1.4112), 0.5246, (0.957)
		a	(1.1301,1.8221), 0.6920, (0.956)	(1.1531,1.7165), 0.5634, (0.961)
	3	λ	(0.8736,1.4087), 0.5351, (0.953)	(0.8809,1.4011), 0.5202, (0.952)
		a	(1.1408,1.7122), 0.5714, (0.961)	(1.1598,1.6987), 0.5389, (0.969)

Table 4. 95% CI for parameters, IL and CP of CI (λ =1.2 , a =1.5, τ=0.3 and η=1.5)

(n,m)	CS	Para.	ACI, IL, CP	BCI, IL, CP
(80,40)	1	λ	(0.8277,1.6471), 0.8194, (0.941)	(0.8437,1.5124), 0.6687, (0.933)
		a	(0.8764,1.9341), 1.0577, (0.958)	(1.0981,1.8987), 0.8006, (0.947)
	2	λ	(0.8365,1.4456), 0.6091, (0.942)	(0.8278,1.4212), 0.5934, (0.967)
		a	(1.0134,1.9016), 0.8882, (0.957)	(1.0198,1.8679), 0.8481, (0.955)
	3	λ	(0.8445,1.4612), 0.6167, (0.952)	(0.8643,1.4501), 0.5858, (0.948)
		a	(0.9487,1.9001), 0.9514, (0.949)	(1.0675,1.8821), 0.8146, (0.961)
(80,60)	1	λ	(0.8398,1.4453) , 0.6055, (0.945)	(0.8403,1.4212), 0.5809, (0.957)
		a	(1.0009,1.9101), 0.9092, (0.954)	(1.0112,1.8567), 0.8455, (0.949)
	2	λ	(0.8478,1.4409), 0.5931, (0.952)	(0.8422,1.4376), 0.5954, (0.955)
		a	(1.0113,1.8147), 0.8034, (0.967)	(1.1167,1.8133), 0.6966, (0.942)
	3	λ	(0.8378,1.4265), 0.5887, (0.948)	(0.8465,1.4267), 0.5802 (0.945)
		a	(1.0568,1.8228), 0.7660, (0.962)	(1.1371,1.8187), 0.6816, (0.953)
(100,40)	1	λ	(0.8456,1.4321), 0.5865, (0.957)	(0.8478,1.4278), 0.5800, (0.961)
		a	(1.1144,1.8765), 0.7621, (0.965)	(1.1231,1.7007), 0.5776, (0.959)
	2	λ	(0.8309,1.4087), 0.5778, (0.962)	(0.8412,1.4076), 0.5664, (0.964)
		a	(1.1012,1.7643), 0.6631, (0.939)	(1.1412,1.6885), 0.5473, (0.957)
	3	λ	(0.8436,1.4301), 0.5865, (0.961)	(0.8615,1.4238), 0.5623, (0.959)
		a	(1.0423,1.7445), 0.7022, (0.954)	(1.1035,1.6756), 0.5721, (0.971)
(100,60)	1	λ	(0.8813,1.4328), 0.5515, (0.955)	(0.8809,1.4167), 0.5358, (0.962)
		a	(1.1167,1.8008), 0.6841, (0.963)	(1.1577,1.7787), 0.6210, (0.965)
	2	λ	(0.8675,1.4212), 0.5537, (0.967)	(0.8712,1.4109), 0.5397, (0.968)
		a	(1.0271,1.8024), 0.7753, (0.962)	(1.1556,1.7298), 0.5742, (0.957)

3	λ	(0.8721,1.4065), 0.5344, (0.952)	(0.8806,1.4002), 0.5196, (0.969)
	a	(1.1306,1.7117), 0.5811, (0.967)	(1.1501,1.6741), 0.5240, (0.971)

From Tables 1, 2, 3 and 4, it may be observed that

(1). For fixed η, τ and n, the MSE of the MLE decreases as m increases, and A-MLE is closer to the real value. For fixed η, τ and m, the MSE of the MLE decreases as n increases.

(2). For fixed η, τ, the MSE of the MLE decreases as n and m increase at the same time.

(3). For fixed n, m and η, the MSE of the MLE decreases as τ decreases. For fixed n, m and τ, the MSE of the MLE decreases as η increases.

(4). The bootstrap confidence interval has the more smaller length and more bigger coverage probabilities than the approximate confidence interval

Thus, the procedure proposed in this paper can achieve good estimation performance.
